# Genomic basis of antibiotic resistance in *Vibrio parahaemolyticus* strain JPA1

**DOI:** 10.1590/0074-02760190053

**Published:** 2019-04-25

**Authors:** Felipe Hernandes Coutinho, Diogo Antonio Tschoeke, Maysa Mandetta Clementino, Cristiane Carneiro Thompson, Fabiano Lopes Thompson

**Affiliations:** 1 Universidade Federal do Rio de Janeiro, Instituto de Biologia, Rio de Janeiro, RJ, Brasil; 2 Fundação Oswaldo Cruz-Fiocruz, Instituto Nacional de Controle de Qualidade Sanitária, Rio de Janeiro, RJ, Brasil; 3 Universidade Federal do Rio de Janeiro, Instituto Alberto Luiz Coimbra de Pós-Graduação e Pesquisa de Engenharia, Laboratório de Sistemas Avançados de Gestão da Produção, Rio de Janeiro, RJ, Brasil

**Keywords:** Vibrio parahaemolyticus, antibiotic resistance, urban lagoon, multi-drug resistance

## Abstract

A multi-resistant strain of *Vibrio parahaemolyticus* was isolated from a tropical estuary in Rio de Janeiro, Brazil. Genome sequencing was conducted to establish the molecular basis of antibiotic resistance in this organism. The genetic content of this strain revealed it to be a non-virulent lineage that nevertheless possesses several antibiotic resistance determinants.

Virulent strains of *Vibrio parahaemolyticus* are responsible for several global outbreaks of gastroenteritis caused by the ingestion of contaminated seafood.^1^ ^,^ ^2^ This organism is typically found in warm aquatic environments and is often associated with invertebrates, either adopting a virulent or non-virulent lifestyle.^3^ ^,^ ^4^ ^,^ ^5^ ^,^ ^6^ Although several antibiotic-resistant strains of *V. parahaemolyticus* have been reported,^4^ ^,^ ^6^ ^,^ ^7^ little has been done to elucidate the genetic basis of resistance among the environmental lineages. To tackle this issue, we isolated a multi-resistant strain of *V. parahaemolyticus*, hereby named strain JPA1, from the waters of the Jacarepaguá lagoon system situated in the city of Rio de Janeiro, Brazil. The local population often comes in contact with the waters at this site either directly for recreational purposes or indirectly through the consumption of seafood retrieved from the lagoon system. Despite this, the Jacarepaguá lagoons receive massive amounts of untreated sewage daily; a factor that contributes to the high abundance and diversity of antibiotic-resistant bacteria in this habitat.^8^ ^,^ ^9^ Therefore, understanding the diversity of the antibiotic-resistant bacteria dwelling in the Jacarepaguá lagoons and their molecular mechanisms of resistance can provide insights into the potential risks that these organisms pose to the local population and elucidate how resistance can spread among aquatic bacteria in this habitat.

The antibiotic susceptibility profile of strain JPA1 was determined by measuring the minimum inhibitory concentration (MIC) of 16 drugs against JPA1. This organism was resistant to eight out of the 16 tested antibiotics (Table I). JPA1 showed resistance or intermediate resistance to all the tested beta-lactams, with the exception of ceftriaxone. However, it tested susceptible to all aminoglycosides, tigecycline, and ciprofloxacin.

**TABLE I:** Antibiotic susceptibility profile of *Vibrio parahaemolyticus* strain JPA1

Antibiotic	Class	MIC (μg/mL)	Phenotype
Ceftriaxone	Beta-Lactam	8	Susceptible
Meropenem	Aminoglycoside	4	Susceptible
Amikacin	Aminoglycoside	16	Susceptible
Gentamicin	Aminoglycoside	2	Susceptible
Ciprofloxacin	Ciprofloxacin	< = 0.25	Susceptible
Tigecycline	Glycycycline	< = 0.5	Susceptible
Piperacillin/Tazobactam	Beta-Lactam	64	Intermediate
Imipenem	Beta-Lactam	8	Intermediate
Ampicillin	Beta-Lactam	> = 32	Resistant
Ampicillin/Sulbactam	Beta-Lactam	> = 32	Resistant
Cefuroxime	Beta-Lactam	> = 64	Resistant
Cefuroxime Axetil	Beta-Lactam	> = 64	Resistant
Cefoxitin	Beta-Lactam	> = 64	Resistant
Ceftazidime	Beta-Lactam	32	Resistant
Cefepime	Beta-Lactam	> = 64	Resistant
Colistin	Polymyxin	4	Resistant

MIC: minimum inhibitory concentration.

DNA was prepared for sequencing using the Nextera XT DNA library prep kit following manufacturer’s recommendations. Genome sequencing was conducted using the Illumina MiSeq platform that yielded 1,461,209 reads (average length = 250 bp and average Phred score = 37). Reads were subjected to a hybrid assembly using A5^10^ and SPAdes.^11^ The 5.1 Mbp draft genome of the *V. parahaemolyticus* strain JPA1 was assembled into 793 scaffolds (N50 = 17,960 bp) and displayed a G+C content of 45.1%. Gene prediction was carried out using Prokka,^12^ and the predicted proteins were annotated using Diamond^13^ for best-hit classification against the NCBI nr database. The assembled genome was deposited in the European Nucleotide Archive under project PRJEB31105.

Clinical strains of *V. parahaemolyticus* often carry genes that encode a type three secretion system (T3SS) for a thermostable direct haemolysin (TDH) and/or TDH-related haemolysin.^1^ ^,^ ^14^ However, neither were detected in the genome of *V. parahaemolyticus* JPA1, suggesting it to be non-virulent to humans. Yet the JPA1 genome encoded genes that were involved in resistance against several classes of antibiotics (Table II). We did not detect these genes in association with any mobile genetic elements, which shows that these are intrinsic resistance mechanisms. Genes coding for three main resistance mechanisms were identified: multi-drug efflux pumps, antibiotic inactivation, and target protection. Efflux pumps confer resistance by pumping antibiotics and other drugs out of the bacterial cytoplasm. Among the efflux pumps identified in the JPA1 genome, those associated with resistance to aminoglycosides, beta-lactams, fluoroquinolones, macrolides, streptogramin, and tetracycline were found. JPA1 also possessed genes for the assembly of the AcrEF-TolC complex, which is a multi-drug efflux pump capable of removing a broad array of drugs from the bacterial cytoplasm. Genes for the MacAB-TolC complex, which grants resistance to macrolides, were also detected. Other cases of efflux pumps conferring resistance are as follows: *novA*, which encodes an ABC type III transporter that confers resistance to novobiocin; *vgaE*, which confers resistance to streptogramin; *tet34* and *tet35*, both of which confer resistance to tetracyclines; and *sav1866*, which encodes a non-specific multi-drug transporter.

**TABLE II:** Antibiotic resistance genes identified in the genome of *Vibrio parahaemolyticus* JPA1

Protein	Gene	Antibiotic	Resistance mechanism
Multidrug export protein AcrE precursor	acrE	Fluoroquinolones,Beta-Lactams	Efflux
Aminoglycoside-3'-phosphotrasnsferase	APH(3'')-Ib	Amynoglicosides	Antibiotic inactivation
Beta-lactamase precursor	CARB-18	Beta-Lactams	Antibiotic inactivation
Chloramphenicol acetyltransferase	catB8	Phenicols	Antibiotic inactivation
cAMP-activated global transcriptional regulator CRP	CRP	Macrolides, Fluoroquinolones, Beta-Lactams	Regulation of efflux
Dihydrofolate reductase type 3	dfrA3	Trimpethoprim	Target replacement
DNA-binding protein H-NS	H-NS	Macrolides, Fluoroquinolones, Beta-Lactams	Regulation of efflux
Macrolide export protein MacA	macA	Macrolides	Efflux
Macrolide export ATP-binding/permease protein MacB	macB	Macrolides	Efflux
Multidrug resistance protein NorM	mdtK	Fluoroquinolones	Efflux
Efflux pump membrane transporter BepE	mexI	Fluoroquinolones	Efflux
Phosphate regulon transcriptional regulatory protein PhoB	PhoB	Multiple	Regulation of efflux
Sensor histidine kinase TodS	TodS	Multiple	Regulation of efflux
Lipid A export ATP-binding/permease protein MsbA	MsbA	Novobiocin	Efflux
GTP-binding protein TypA/BipA	otrA	Oxytetracycline	Target protection protein
UDP-glucose 6-dehydrogenase	PmrE	Polymixin	Target alteration
Hypothetical protein	QnrC	Quinolones	Target protection protein
Secreted effector protein pipB2	QnrVC5	Quinolones	Target protection protein
Putative multidrug export ATP-binding/permease protein	sav1866	Multiple	Efflux
Elongation factor G 1	tet32	Tetracyclines	Target protection protein
Xanthine phosphoribosyltransferase	tet34	Tetracycline	Efflux
Malate-2H(+)/Na(+)-lactate antiporter	tet35	Tetracycline	Efflux
Elongation factor G	tetW	Tetracyclines	Target protection protein
Outer membrane protein TolC precursor	tolC	Multiple	Efflux
Putative ABC transporter ATP-binding protein YjjK	vgaE	Streptogramin	Efflux

Three genes encoding proteins capable of antibiotic inactivation were also detected: *APH(3’’)-Ib* encodes an aminoglycoside-3’-phosphotransferase capable of inactivating aminoglycosides through phosphorylation, *CARB-18* encodes a β-lactamase, and *catB8* encodes a chloramphenicol acetyltransferase that inactivates amphenicols. Target protection proteins work by impairing the contact between antibiotics and their targets. Gene *dfrA3* encodes an alternative dihydrofolate reductase that is less sensitive to the action of trimethoprim. Genes *otrA, tetW*, and *tet32* encode peptides that perform non-covalent modifications to bacterial ribosomes, rendering them resistant to tetracyclines. Furthermore, *qnrC* and *qnrVC5* also contribute to target protection mechanisms that confer resistance to quinolones.

Upon infection, human pathogens are often challenged by antibiotic therapy, which favours strains that possess antibiotic resistance determinants. Many potentially pathogenic bacteria possibly have a free-living lifestyle that includes surviving in soils, water bodies, and associated to non-human hosts. JPA1’s genetic content indicates that it is non-pathogenic to humans, despite possessing a broad array of antibiotic resistance genes. Antibiotic resistance genes precede the advent of antibiotic therapy,^15^ ^,^ ^16^ which indicates that these genes may play a different role in bacterial physiology under non-clinical settings.^17^ This particularly explains the prevalence of antibiotic resistance genes in the JPA1 genome.

Thus, we conclude that the JPA1 genome has a broad array of antibiotic resistance genes that confer upon it a multi-resistant phenotype. Horizontal gene transfer has been implicated as a mechanism for the acquisition of virulence^18^ and antibiotic resistance^19^ genes in *V. parahaemolyticus*. In the environment, horizontal gene transfer is often mediated by plasmids and other mobile genetic elements. However, the resistance mechanisms identified in the JPA1 genome were not found to be associated with such elements. Nevertheless, horizontal gene transfer can also take place through the direct uptake of exogenous DNA or via phage-mediated transduction. In the advent that antibiotic resistance genes from JPA1 are mobilised to other bacteria through the aforementioned mechanisms, this strain could play a role in the spread of antibiotic resistance genes in aquatic ecosystems.^6^ This is of particular importance considering that JPA1 shares its habitat with many potentially pathogenic organisms that are medically relevant such as *Vibrio cholerae*, *Shigella* spp., and *Pseudomonas aeruginosa*.^9^
